# An enzyme‐linked immunosorbent assay (ELISA)‐based activity assay for AMP‐activated protein kinase (AMPK)

**DOI:** 10.1002/2211-5463.70017

**Published:** 2025-03-11

**Authors:** Trezze P. Nguyen, Shangze Lyu, Yang Liu

**Affiliations:** ^1^ Department of Integrative Biology and Pharmacology, McGovern Medical School University of Texas Health Science Center at Houston TX USA; ^2^ Molecular and Translational Biology Program MD Anderson Cancer Center UTHealth Graduate School of Biomedical Sciences Houston TX USA

**Keywords:** AMPK, ELISA, kinase activity

## Abstract

AMP‐activated protein kinase (AMPK) is the master regulator of cellular and organismal energy homeostasis, playing an essential role in modulating metabolism and other cellular processes. Substantial efforts have been made to develop pharmacological modulators of AMPK activity due to their therapeutic potential against various diseases. Measuring AMPK activity *in vitro* is a fundamental step for testing AMPK activators and inhibitors. Here, we report an enzyme‐linked immunosorbent assay (ELISA)‐based AMPK activity assay with simple steps and high sensitivity. This assay offers a robust, in‐house alternative to the traditional radioactive methods and other approaches that rely on specialized reagents or commercial kits.

## Graphical abstract

Measuring AMP‐activated protein kinase (AMPK) activity *in vitro* is crucial for testing AMPK activators or inhibitors with therapeutic potential. Here, we report an enzyme‐linked immunosorbent assay (ELISA)‐based AMPK activity assay with simple steps and high sensitivity, which offers a simple, robust, and cost‐effective alternative to traditional radioactive assays and other methods that rely on specialized reagents or commercial kits.

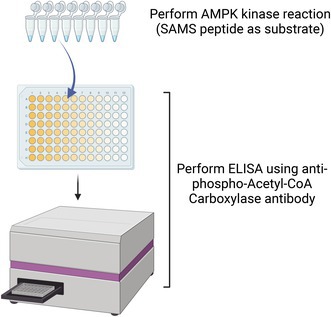

**Abbreviations**
ADaM: allosteric drug and metabolite
ADP: adenosine diphosphate
AMP: adenosine monophosphate
AMPK: AMP‐activated protein kinase
ATP: adenosine triphosphate
CaMKK2: calcium/calmodulin‐dependent protein kinase kinase 2
ELISA: enzyme‐linked immunosorbent assay
LKB1: liver kinase B1
OD: optical density

AMP‐activated protein kinase (AMPK) plays an essential role in maintaining cellular and organismal energy homeostasis [1]. It is activated when the cellular ATP levels decrease and AMP and ADP levels increase [1]. Upon activation, AMPK restores cellular energy balance by phosphorylating various downstream substrates, leading to the suppression of energy‐intensive anabolic pathways, such as protein and fatty acid synthesis, while enhancing energy‐producing catabolic processes, including glycolysis and fatty acid oxidation [2]. In addition to its metabolic functions, AMPK plays a broader role in regulating other key cellular processes, including autophagy, mitochondrial dynamics, lysosomal function, and DNA repair [1, 3]. Given its central roles in maintaining cellular energy homeostasis and regulating multiple processes, AMPK has emerged as an attractive therapeutic target. Pharmacological modulators of AMPK, particularly activators, are being explored for their therapeutic potential in treating a variety of pathological conditions, particularly metabolic diseases and syndromes [4].

AMPK is a heterotrimeric complex containing one catalytic subunit (α) and two regulatory subunits (β and γ). In mammalian cells, there are two α isoforms (α1 and α2), two β isoforms (β1 and β2), and three γ isoforms (γ1, γ2 and γ3), which give rise to 12 different heterotrimeric combinations. Certain heterotrimers may be dominantly expressed in specific tissues or cell types [1]. AMPK can be activated through phosphorylation of a threonine residue (Thr172) on the activation loop of the α subunit by upstream kinases such as LKB1 and CaMKK2 [1]. In addition, AMPK can also be activated allosterically. For example, AMP binds to the γ subunit, which leads to allosteric activation of AMPK [5]. Additionally, AMP binding to AMPK promotes the phosphorylation of Thr172 of the α subunit by the upstream kinases and protects this residue from dephosphorylation [5, 6]. Moreover, many direct allosteric activators of AMPK discovered in recent years bind to the ADaM (Allosteric Drug and Metabolite) site, which forms between the carbohydrate‐binding (CBM) domain of the β subunit and the N‐lobe of the α subunit kinase domain [4, 7]. Some of these activators have shown great therapeutic potential in treating metabolic diseases and syndromes such as diabetes and NASH (nonalcoholic steatohepatitis) [8, 9].

Measuring AMPK kinase activity *in vitro* is an essential step for identifying small‐molecule AMPK activators and inhibitors as well as understanding the regulatory mechanisms underlying AMPK activation. The classic methods use radiolabeled ^32^P‐ or ^33^P‐ATP in the kinase reaction to track the incorporation of ^32^P or ^33^P into the kinase substrate peptides. However, these methods involve handling radioactive materials, which pose safety and disposal challenges. In recent years, different nonradioactive methods have been proposed, including several FRET (Fluorescence Resonance Energy Transfer)‐based assays [10, 11] that measure the amount of phosphorylated substrates in a kinase reaction as well as other assays that measure ATP consumption during the kinase reaction [12]. These methods often require specialized reagents such as fluorescent dye‐ or biotin‐labeled substrate peptides or/and antibodies as well as commercial kits. Here, we report a highly sensitive ELISA‐based AMPK activity assay using commonly available reagents without the need for specialized materials. We applied this method to calculate the kinetic parameters of recombinant AMPK complexes and evaluate the effects of known small‐molecule modulators. This assay represents a practical and cost‐effective tool for researchers studying AMPK, offering a reliable alternative to commercial kits and radioactive approaches.

## Materials and methods

### Reagents

Recombinant AMPK complexes α1β1γ1 and α2β1γ1 (N‐terminally His‐tagged) were expressed in *Escherichia coli* and purified using nickel affinity chromatography and gel filtration as described previously [11]. AMPK complexes were phosphorylated on Thr172 of the α subunit by incubation with Mg.ATP and recombinant CaMKK2 overnight at 18 °C and further purified with a final gel filtration step. A stock solution of the activated AMPK complex was prepared at around 5 mg·mL^−1^ in 50 mm Tris (pH 8.0), 300 mm NaCl, and 1 mm Tris(2‐carboxyethyl)phosphine for further usage. AMPK substrate, SAMS peptide (sequence: HMRSAMSGLHLVKRR), and AMP solution were purchased from SignalChem Biotech (Richmond, BC, Canada; cat# S07‐58 and cat# A46‐09‐500, respectively). Phospho‐SAMS peptide (sequence: HMRSAMpSGLHLVKRR) was synthesized by Alan Scientific (Gaithersburg, MD, USA). ATP solution was purchased from New England Biolabs (Ipswich, MA, USA; cat# P0756). Anti‐phospho‐Acetyl‐CoA carboxylase (Ser79) antibody, anti‐AMPK β1 antibody, and Horseradish peroxidase (HRP)‐conjugated donkey anti‐rabbit IgG antibody were purchased from Millipore Sigma (Burlington, MA, USA; cat# 07‐303, cat# SAB5700079 and cat# AP182P, respectively). One‐Step Ultra TMB ELISA substrate solution was purchased from Thermo Fisher Scientific (Waltham, MA, USA; cat# 34028). AMPK inhibitor, compound C (cat# AOB2281), and AMPK activators, compound 991 (cat# AOB8150) and PF‐739 (cat# AOB33584), were purchased from AOBIOUS (Gloucester, MA, USA). Dynabeads™ Protein G was purchased from Invitrogen (Carlsbad, CA, USA; cat# 10003D). Protease inhibitor cocktail was purchased from Roche (Basel, Switzerland; cat# 11836170001). Normal rabbit IgG was purchased from Cell Signaling Technology (Danvers, MA, USA; cat# 2729).

### AMPK activity assay using ELISA

#### AMPK kinase reaction with recombinant AMPK

**Fig. 1:**
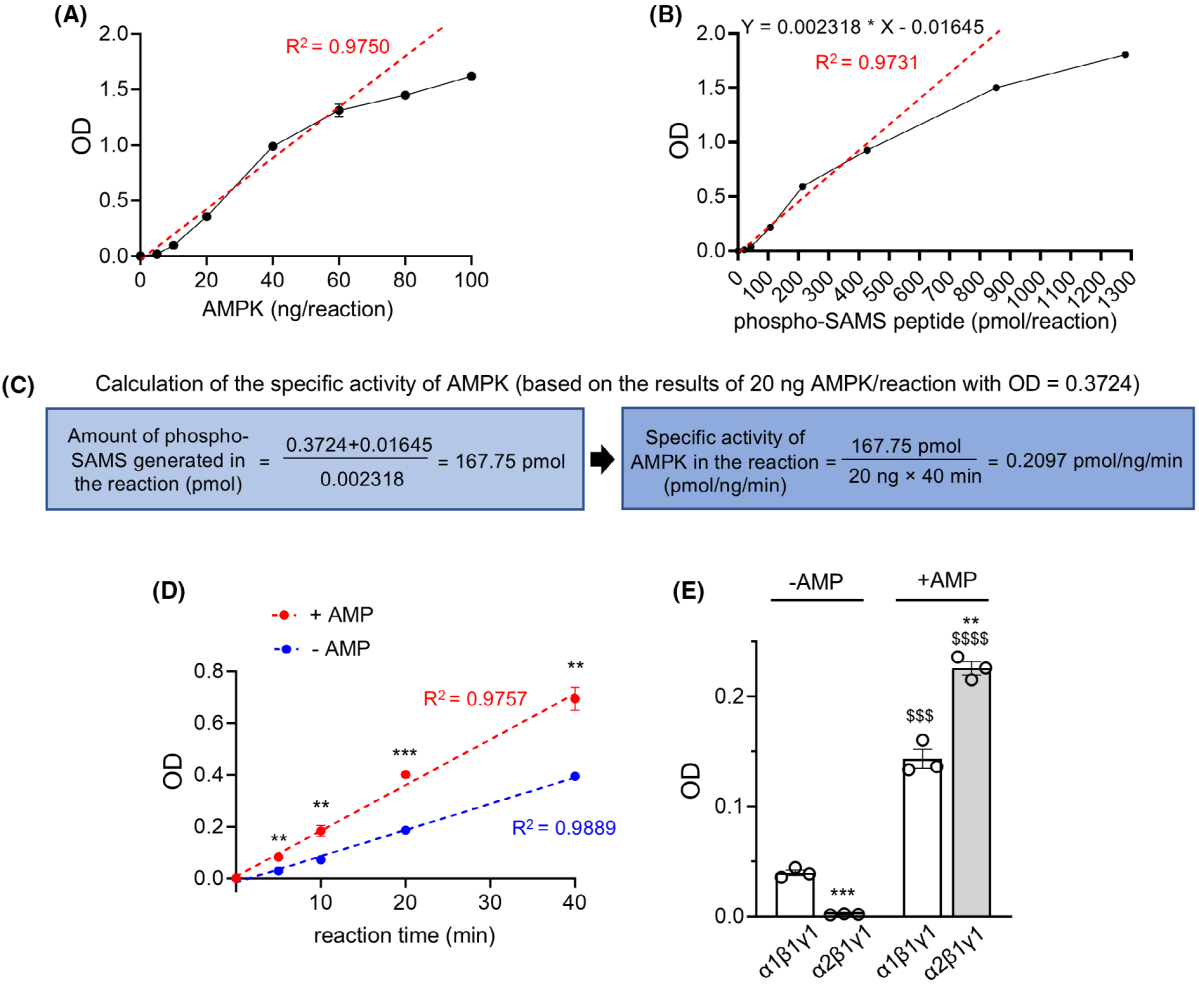
Validation of the proposed AMPK activity assay. (A) OD values from the ELISA following the kinase reactions (40‐min reaction time) with different amounts of AMPK α1β1γ1 (0, 5, 10, 20, 40, 60, 80 or 100 ng per reaction), *n* = 4. OD values from the condition of 0 ng AMPK were considered as blank values and subtracted from all the OD values in other conditions. *R* ^2^ was calculated based on the data from conditions with 0, 5, 10, 20, 40, and 60 ng AMPK/reaction (red dotted line). (B) A standard curve established using different concentrations of phospho‐SAMS peptide (0, 21.34, 42.69, 106.72, 213.43, 426.86, 853.72, or 1280.58 pmol/reaction), *n* = 3. OD values from the condition of 0 pmol/reaction were considered as blank values and subtracted from all the OD values in other conditions. *R* ^2^ and the equation between *Y* (OD value) and *X* (pmol phospho‐SAMS peptide/reaction) were established based on the data from the conditions with 0, 21.34, 42.69, 106.72, 213.43, and 426.86 pmol phospho‐SAMS peptide/reaction (red dotted line). (B) was done in the same experiment as shown in (A). (C) Specific activity of AMPK α1β1γ1 based on the condition of 20 ng AMPK/reaction shown in (A) (OD = 0.3724) was calculated using the equation established in (B). (D) OD values from the ELISA following 0, 5, 10, 20, and 40 min of kinase reactions with 20 ng AMPK α1β1γ1 per reaction in the presence or absence of AMP, *n* = 3. OD values from the condition of 0‐min reaction were considered as blank values and subtracted from all the OD values in other conditions. *R* ^2^ was calculated based on the data from all the time points. **P < 0.01, ***P < 0.001 when comparing the +AMP condition to the −AMP condition with the same reaction time by Student's *t*‐test. (E) OD values from the ELISA following 20 min of kinase reactions with 20 ng AMPK α1β1γ1 or AMPK α2β1γ1 per reaction in the presence or absence of AMP, *n* = 3. OD values from the condition of 0‐min reaction were considered as blank values and subtracted from all the OD values in other conditions. **P < 0.01, ***P < 0.001 when comparing α2β1γ1 to α1β1γ1 with the same AMP availability in the reaction using Student's *t*‐test. ^$$$^ *P* < 0.001, ^$$$$^ *P* < 0.0001 when comparing to the same heterotrimer in the absence of AMP using Student's *t*‐test. Data are mean ± SEM. AMP, adenosine monophosphate; AMPK, AMP‐activated protein kinase; ELISA, enzyme‐linked immunosorbent assay; OD, optical density.

AMPK kinase reactions were performed in PCR tubes. For each kinase reaction (20 μL), 4 μL of 5× kinase buffer (200 mm Tris–HCl, pH 7.4, 100 mm MgCl_2_, 0.5 mg·mL^−1^ BSA and 0.25 mm DTT), 50 μm AMP, 20 ng recombinant AMPK (α1β1γ1 or α2β1γ1), 0.2 μg·μL^−1^ (112 μm) SAMS peptide, 500 μm ATP, and H_2_O (molecular biology level; to make the final volume 20 μL) were added. In some experiments, AMP was excluded from the reactions, and variable concentrations of AMPK, SAMS peptide, and ATP were used as indicated in the text or figure legends. In the experiment when the AMPK inhibitor (compound C) and AMPK activators (compound 991 and PF‐739) were tested, the stock solutions of the inhibitor and activators (all dissolved in DMSO) as well as DMSO (as control) were properly prepared and added into the kinase reaction mixture to make the final concentration of DMSO 0.05% (v/v). ATP was added lastly to start the kinase reactions, which were carried out at room temperature for the period of time as indicated in the figure legends. When multiple reaction time points were studied, as shown in Fig. 1D, after reaching one desired time point, 4.5 μL of the reaction mixture was taken out and mixed with 4.5 μL 50 mm EDTA to stop the kinase reaction, and this sample harvesting process was repeated for the desired time points. If only one reaction period was used in the experiments, when reaching the time point, 20 μL 50 mm EDTA was added to the reaction mixture to stop the kinase reaction. To make the blank control without kinase reaction, the kinase mixture without ATP was directly added to 20 μL 50 mm EDTA before ATP was added lastly. To make a standard curve for measuring the exact amount of phosphorylated SAMS peptide after each kinase reaction, a series of peptide mixtures with a constant total peptide concentration of 200 ng·μL^−1^ but different proportions of SAMS peptide and phospho‐SAMS peptide were made in 1x kinase buffer. Specifically, a series of mixtures containing 0 ng·μL^−1^ (0 pmol per 20 μL), 2 ng·μL^−1^ (21.34 pmol per 20 μL), 4 ng·μL^−1^ (42.69 pmol per 20 μL),10 ng·μL^−1^ (106.72 pmol per 20 μL), 20 ng·μL^−1^ (213.43 pmol per 20 μL), 40 ng·μL^−1^, (426.86 pmol per 20 μL), 80 ng·μL^−1^ (853.72 pmol per 20 μL), and 120 ng·μL^−1^ (1280.58 pmol per 20 μL) of phospho‐SAMS peptide (with variable amounts of SAMS peptide to make the total concentration of peptide 200 ng·μL^−1^) were made, and then 20 μL of these mixtures were mixed with 20 μL 50 mm EDTA.

#### AMPK reaction with AMPK β1 immunoprecipitants from cells

Hek293 cells (ATCC, Manassas, VA, USA) were cultured in DMEM with 10% FBS. Half a million cells were seeded in each well of the 12‐well plate 24 h before being subjected to a 2‐h treatment with 1 μm compound 991 or DMSO as control. After the treatment, 300 μL lysis buffer (50 mm Hepes pH 7.4, 50 mm KCl, 50 mm NaF, 5 mm Na_4_P_2_O_7_, 5 mm β‐glycerophosphate, 1 mm EDTA, 1 mm EGTA, 1 mm Na_3_VO_4_, 1 mm dithiothreitol supplemented with protease inhibitor cocktail and 0.2% [v/v] Triton X‐100) was added to each well to prepare the cell lysate. The cell lysate from each well was centrifuged at 16 000 ***g***, 4 °C before the supernatant was taken out and mixed with 1.5 μg anti‐AMPK β1 antibody or normal rabbit IgG (as blank control) plus 30 μL Dynabeads for 2 h on a rotator at 4 °C. After the incubation, the beads were washed three times with lysis buffer and additional two times with 1× kinase buffer before being resuspended in 20 μL 1× kinase buffer. Kinase reactions were carried out with one quarter of the beads obtained (5 μL), 3 μL 5× kinase buffer, 50 μm AMP, 0.2 μg·μL^−1^ (112 μm) SAMS peptide, 500 μm ATP, and H_2_O (molecular biology level; to make the final volume 20 μL) in PCR tubes at room temperature for 40 min. After 40 min, 20 μL 50 mm EDTA was added to the reaction mixture to stop the kinase reaction.

#### ELISA

The stopped reaction mixtures, as well as the blank and standard curve samples, were further diluted five times with peptide coupling buffer (100 mm carbonate buffer, pH 9.6) before 5 μL diluted mixture from each reaction was added into one well of the Nunc Immobilizer Amino microplate (Thermo Fisher Scientific, Waltham, MA, USA; cat# 436013) prefilled with 95 μL peptide coupling buffer. The microplate was incubated either at 4 °C overnight or at room temperature for 2 h with gentle agitation, allowing the peptides from the reaction mixtures to covalently couple to the well surfaces. After the coupling process, the solutions in the wells were dumped out, and the wells were washed three times with TBST (Tris‐buffered saline with 0.1% [v/v] Tween 20; 300 μL/well/wash). The wells were then blocked with blocking buffer containing 1% (w/v) BSA in TBST (100 μL/well) for 1 h at room temperature with gentle agitation. The blocking buffer was then dumped out, and 70 μL primary antibody solution containing anti‐phospho‐Acetyl‐CoA carboxylase (Ser79) antibody diluted in blocking buffer (1 : 200 dilution) was added into each well for 1 h at room temperature. After the incubation with primary antibody, the plate was washed six times with TBST (300 μL/well/wash) before 100 μL secondary antibody solution containing Horseradish peroxidase (HRP)‐conjugated anti‐rabbit IgG antibody diluted in blocking buffer (1 : 5000 dilution) was added into each well for another 1 h at room temperature. The plate was then washed six times with TBST (300 μL/well/wash), followed by the addition of 50 μL of One‐Step Ultra TMB ELISA substrate solution to each well. The plate was incubated for 10–20 min, protected from light. When a desired blue color in each well had appeared, 50 μL ELISA stop solution (0.16 m sulfuric acid) was added per well to stop the enzyme reaction. The plate was then read in a plate reader to measure optical density (OD) at 450 and 650 nm (as background wavelength correction). The OD450 value in each well was adjusted by subtracting the OD650 value before data analysis.

### Data analysis

The corrected OD450 value from each kinase reaction was further normalized by subtracting the blank value before being plotted for comparison. For the standard curve, only data points from the linear portion of the curve were used to establish the *R* ^2^ value, and the equation for converting OD values into the amounts of phospho‐SAMS peptide was determined using the linear regression analysis function in graphpad prism 9.3.1 (GraphPad Software, Boston, MA, USA). For Michaelis–Menten curves, *K*_m_ values were calculated using nonlinear regression–enzyme kinetics analyzing functions in graphpad prism 9.3.1. Graph drawing and statistical analysis were performed with graphpad prism 9.3.1. Student's *t*‐test was used to compare differences between conditions.

## Results and Discussion

The aim of this work was to develop an ELISA‐based assay to measure AMPK activity by quantifying the levels of phosphorylated SAMS peptide (an AMPK substrate peptide derived from Acetyl‐CoA carboxylase) after kinase reaction *in vitro*. In a kinase reaction, AMPK phosphorylates SAMS peptide to generate phospho‐SAMS peptide. The amount of phospho‐SAMS peptide generated during the reaction reflects AMPK kinase activity. To quantify the levels of phospho‐SAMS peptide in different reactions, the reaction mixtures are added into Nunc Immobilizer Amino microplate wells. The surfaces of these wells contain electrophilic groups that covalently conjugate to the nucleophilic primary amines on both phosphorylated and nonphosphorylated SAMS peptides. The levels of phospho‐SAMS peptide in each well are then detected via ELISA using an anti‐phospho‐Acetyl‐CoA carboxylase (Ser79) antibody, which recognizes the phospho‐SAMS peptide. The optical density (OD) signal correlates with the amount of phospho‐SAMS peptide generated in the kinase reaction, reflecting AMPK activity, provided the ELISA signal is within a nonsaturated range. In the experimental process, the coupling of phospho‐SAMS peptide to the surface of the microplate well is an essential step that can be affected by factors such as the kinase buffer of choice. Therefore, this step requires optimization by the end users to determine how much of the kinase reaction mixture should be used for coupling. The experimental procedure described in this study was optimized based on the kinase buffer we chose that contains Tris–HCl and BSA, both of which have primary amines that could potentially compete with SAMS peptide for coupling, although we did not observe interference from these two components on the experimental results we obtained.

To optimize kinase reaction conditions and avoid ELISA signal saturation, we tested varying amounts of AMPK α1β1γ1 in 40‐min reactions with the presence of AMP (50 μm), where SAMS peptide (112 μm) and ATP (500 μm) were at saturated concentrations based on the results from previous studies [13]. ELISA signals remained linear (*R* ^2^ = 0.9750) for AMPK concentrations up to 60 ng/reaction (~ 21 nm) (Fig. 1A). Based on this, we selected 20 ng (~ 6.9 nm) AMPK per reaction for subsequent experiments. If higher AMPK concentrations are needed, end users should reduce the reaction time and verify signal linearity. To calculate the specific activity of AMPK based on the OD values, in the same experiment of Fig. 1A, we also generated a standard curve with defined amounts of phospho‐SAMS peptide/reaction (Fig. 1B), and found that the signal remained in a linear range (*R* ^2^ = 0.9731) with up to 427 pmol phospho‐SAMS peptide/reaction. Within this linear range, an equation, *Y* (OD value) = 0.002318*X (pmol of phospho‐SAMS peptide/reaction) − 0.01645, was derived, which can be used to quantify phospho‐SAMS peptide level (pmol) in each reaction based on the OD value. Using this equation, we calculated the specific activity of AMPK based on the result of the 20 ng AMPK/reaction (OD = 0.3724) in Fig. 1A, and obtained 0.2097 pmol·ng^−1^·min^−1^, indicating that 0.2097 pmol SAMS peptide was phosphorylated by 1 ng AMPK per minute (Fig. 1C). This value aligns with previous studies (~ 0.7 pmol·ng^−1^·min^−1^) using a radioactive assay with similarly prepared AMPK complexes [14]. Due to variability in ELISA signals across experiments, if specific activity needs to be calculated, a standard curve needs to be included in the same experiment to have accurate results. For the same reason, the OD values from different experiments should not be directly compared.

To further validate the method, we performed the kinase reaction (with 20 ng of AMPK α1β1γ1) for different periods of time in the presence or absence of AMP before ELISA for determining the relative amounts of phospho‐SAMS peptide generated in the reactions (Fig. 1D). As expected, we observed a linear correlation between the reaction time and OD value (*R* ^2^ = 0.9757 and 0.9889 for with AMP and without AMP conditions, respectively) with higher OD values observed in the reactions with the presence of AMP. In addition, we used the method to compare the kinase activities of recombinant AMPK α1β1γ1 and α2β1γ1 in the presence or absence of AMP (20 ng recombinant protein per reaction) (Fig. 1E), and found that the kinase activity of α2β1γ1 was significantly lower than that of α1β1γ1 in the absence of AMP but became significantly higher in the presence of AMP. These results align with observations from earlier studies indicating that compared to α1‐containing AMPK heterotrimers, α2‐containing heterotrimers exhibit lower baseline specific activity but demonstrate higher sensitivity to nucleotide activation, such as by AMP [7]. The results above demonstrate the validity as well as the adequate accuracy and sensitivity of the method.

**Fig. 2:**
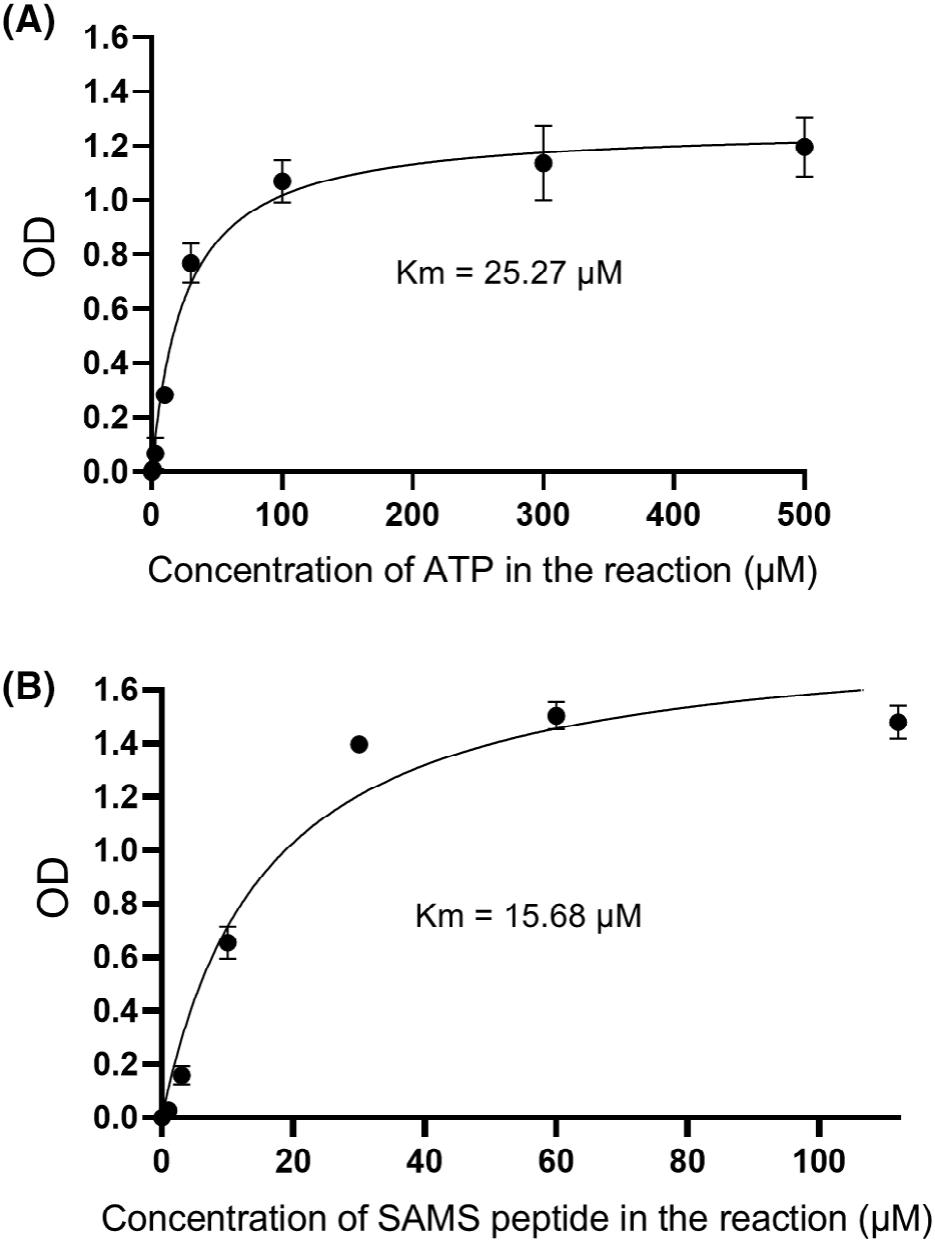
Enzymatic kinetics of AMPK determined by the proposed activity assay. (A) Michaelis–Menten curve based on the concentrations of ATP (0, 1, 3, 10, 30, 100, 300, or 500 μm) in the reaction (40‐min reaction time) and the related OD values, *n* = 3. Values from the condition of 0 μm ATP were considered as blank values and subtracted from all the OD values in other conditions. (B) Michaelis–Menten curve based on the concentrations of SAMS peptide (0, 1, 3, 10, 30, 60, 112 μm) in the reaction (40‐min reaction time) and the related OD values, *n* = 4. Values from the condition of 0 μm SAMS peptide were considered as blank values and subtracted from all the OD values in other conditions. *K*_m_ values in (A) and (B) were calculated using the nonlinear regression‐enzyme kinetics analyzing function in graphpad prism 9.3.1. Data are mean ± SEM. AMPK, AMP‐activated protein kinase; ATP, adenosine triphosphate; OD, optical density.

In addition, we also applied the method to determine the enzymatic kinetics of AMPK α1β1γ1 using different concentrations of the two substrates, ATP and SAMS peptide (Fig. 2A,B). Michaelis–Menten curves were generated using substrate concentrations and the corresponding OD values, allowing for the calculation of *K*_m_ values. From these experiments, we obtained *K*_m_ values of 25.27 μm for ATP and 15.68 μm for SAMS peptide. These values are consistent with those reported in a previous study employing a radioactive method (26.04 μm for ATP and 26.67 μm for SAMS peptide) using similarly prepared AMPK complexes [13]. These results further prove the reliability of this method.

**Fig. 3:**
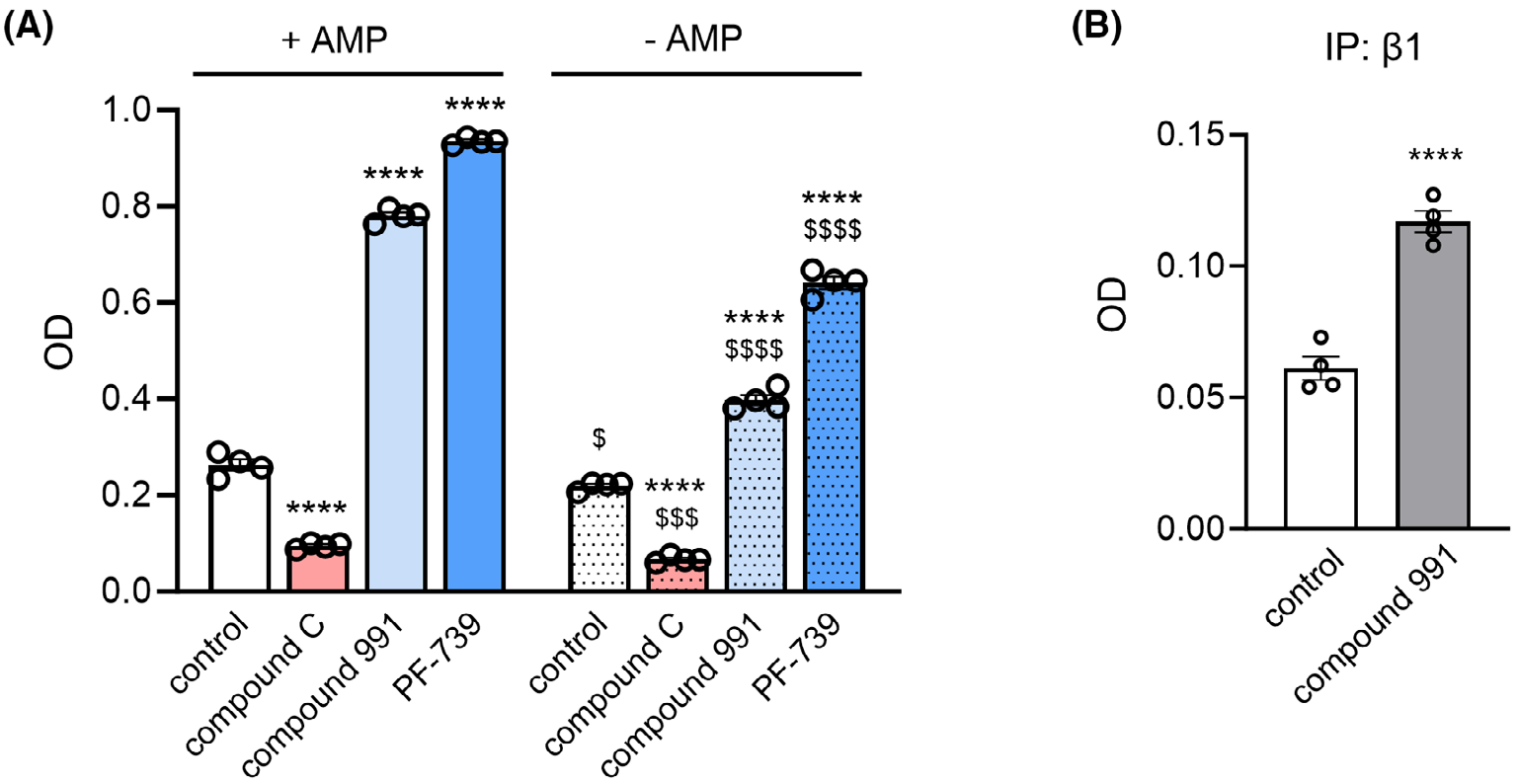
Effects of AMPK activity modulators determined by the proposed activity assay. (A) Compound C (5 μm; an AMPK inhibitor), compound 991 (250 nm; an AMPK activator), PF‐739 (250 nm; an AMPK activator) or DMSO control were added to the AMPK α1β1γ1 kinase reactions (40‐min reaction time) in the presence or absence of AMP. OD values from the ELISA following the kinase reactions were measured and compared, *n* = 4. For each treatment condition, the OD values from 0‐min reaction were considered as blank values and subtracted from the OD values of the experimental conditions. ****P < 0.0001 when comparing to the control condition with the same AMP availability in the reaction using Student's *t*‐test. ^$^ *P* < 0.05, ^$$$^ *P* < 0.001, ^$$$$^ *P* < 0.0001 when comparing to the condition with the same modulator treatment in the presence of AMP using Student's *t*‐test. (B) OD values from the ELISA following the kinase reactions measuring the activity of AMPK β1 immunoprecipitants purified from Hek293 cells treated with 1 μm compound 991 or DMSO (as control) for 2 h, *n* = 4. OD values of the conditions with control IgG immunoprecipitants were considered as blank values and subtracted from the OD values of the experimental conditions. ****P < 0.0001 when comparing the compound 991 treatment condition to the control condition using Student's *t*‐test. Data are mean ± SEM. AMP, adenosine monophosphate; AMPK, AMP‐activated protein kinase; ELISA, enzyme‐linked immunosorbent assay; OD, optical density.

Next, we employed the method to examine the effects of several known AMPK activity modulators, including one inhibitor, compound C [15], and two direct allosteric activators, compound 991 [16, 17] and PF‐739 [8], on AMPK α1β1γ1 kinase activity in the presence or absence of AMP in the reaction (Fig. 3A). As expected, compound C treatment significantly reduced AMPK kinase activity (by ~ 64% and 69% in conditions with and without AMP, respectively), while compound 991 or PF‐739 treatment significantly increased the AMPK kinase activity in both conditions with and without AMP (for compound 991, ~ 3.0 and 1.8 fold increases in the conditions with and without AMP, respectively; for PF‐739, ~ 3.6 and 2.9 fold increases in the conditions with and without AMP, respectively). In all the treatment conditions, the AMPK activity was higher with the presence of AMP in the reaction. We further applied this method to measure the kinase activity of AMPK β1 immunoprecipitants isolated from cells treated with or without compound 991. As expected, β1 immunoprecipitants from cells treated with compound 991 showed significantly higher kinase activity than those from untreated control cells (Fig. 3B), consistent with previous findings [17]. These results demonstrate the applicability of this method for evaluating the effects of AMPK modulators, either *in vitro* using recombinant AMPK complexes or in cell‐based studies using AMPK immunoprecipitants.

## Conclusions

Our results denoted the great feasibility, reliability, and sensitivity of the ELISA‐based AMPK activity assay described in this study. This simple, in‐house method represents an alternative to the traditional radioactive methods, as well as other techniques requiring special reagents and commercial kits, and can be applied to identify and test AMPK modulators for pharmaceutical purposes.

## Conflict of interest

The authors declare no conflict of interest.

### Peer review

The peer review history for this article is available at https://www.webofscience.com/api/gateway/wos/peer‐review/10.1002/2211‐5463.70017.

## Author contributions

TPN and SL carried out experiments and collected the data. YL designed the experiments, analyzed the data, and prepared the manuscript. All authors reviewed and approved the final article version.

## Data Availability

All the data are contained in the manuscript. The original source data for each figure of this study are available from the corresponding author upon reasonable request.
